# 50 shades of oxidative stress: A state-specific cysteine redox pattern hypothesis

**DOI:** 10.1016/j.redox.2023.102936

**Published:** 2023-10-17

**Authors:** James N. Cobley

**Affiliations:** Cysteine redox technology Group, Life Science Innovation Centre, University of the Highlands and Islands, Inverness, IV2 5NA, Scotland, UK

**Keywords:** Oxidative stress, Redox-regulation, Cysteine, Oxidation, ROS, Oxiform, Technology, Pattern

## Abstract

Oxidative stress is biochemically complex. Like primary colours, specific reactive oxygen species (ROS) and antioxidant inputs can be mixed to create unique “shades” of oxidative stress. Even a minimal redox module comprised of just 12 (ROS & antioxidant) inputs and 3 outputs (oxidative damage, cysteine-dependent redox-regulation, or both) yields over half a million “shades” of oxidative stress. The present paper proposes the novel hypothesis that: state-specific shades of oxidative stress, such as a discrete disease, are associated with distinct tell-tale cysteine oxidation patterns. The patterns are encoded by many parameters, from the identity of the oxidised proteins, the cysteine oxidation type, and magnitude. The hypothesis is conceptually grounded in distinct ROS and antioxidant inputs coalescing to produce unique cysteine oxidation outputs. And considers the potential biological significance of the holistic cysteine oxidation outputs. The literature supports the existence of state-specific cysteine oxidation patterns. Measuring and manipulating these patterns offer promising avenues for advancing oxidative stress research. The pattern inspired hypothesis provides a framework for understanding the complex biochemical nature of state-specific oxidative stress.

## Graphical abstract

**Figure:**
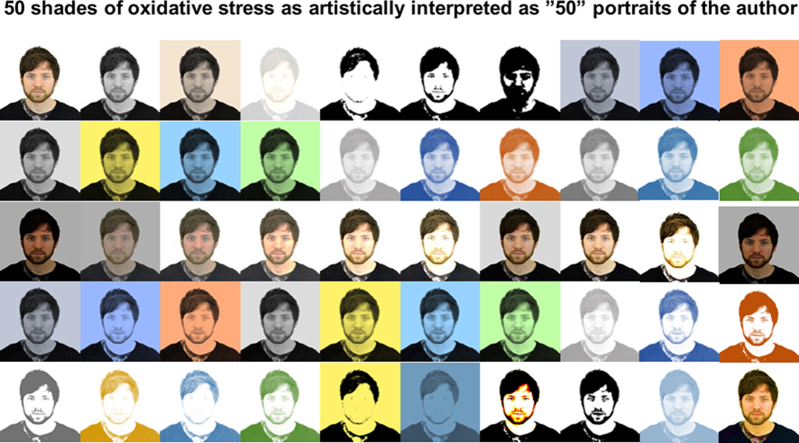

## 1 Introduction

Where there is oxygen, be it the diatomic gas in air or an atom bonded to protons in water, there will inevitably be oxygen-derived free radicals and related non-radicals [[1], [2], [3], [4]], collectively termed reactive oxygen species (ROS) [5,6]. In the wake of Gerschman and Gilbert [7] proposing oxygen poisoning (radicals from air-derived oxygen [8]) and radiation toxicity (radicals from water [9]) sharing a free radical mechanism in common, Harman [10] suggested ROS cause ageing in 1956. And so, before the field fully accepted that cells produce ROS when McCord and Fridovich [11] discovered superoxide dismutase in 1969, ROS were cast as villains. Their villainous character arose from perpetrating harm, such as oxidising DNA [12]. In 1985, Sies captured the “first age” of redox research when he coined oxidative stress as an imbalance between ROS and antioxidants, in favour of the former, that results in oxidative damage [13]. He lucidly defined a crisp input and output relationship:

ROS > antioxidants = oxidative stress (e.g., ↑ oxidative damage).

Unexpected plot twists challenged the myopic historical view of ROS in the “second age” of redox research. Seeds sown in the 1960–1980's [[14], [15], [16], [17], [18], [19]] bore fruit in the 1990's when they ushered in a ground-breaking conceptual revolution [[20], [21], [22]]. Like a good spy, ROS are classic double-agents [23]. Seminal studies, particularly on growth-factor signalling [[24], [25], [26]], unmasked their Janus-face. They laid the foundations of the modern consensus that ROS play multiple beneficial roles via a pervasive evolutionary conserved mechanism: cysteine-dependent redox-regulation [[27], [28], [29], [30], [31], [32], [33]]. Cysteine-dependent redox-regulation defined a new oxidative stress [[34], [35], [36]] output:

ROS >/∼ antioxidants = redox-regulation.

At the dawn of a “third age” in redox research, oxidative stress looks set evolve to a mobile element oscillating about a “good” and “bad” eustress-distress outcome spectrum [37,38]. The eustress-distress spectrum implies there is always oxidative stress of one form or another. One form or another muddies the waters when it comes to arguably the ultimate goal of redox research in any age: to unmask the biological roles of ROS [39]. It implies that one must measure and manipulate specific forms of oxidative stress. But, are there specific forms? If so, how many forms are there? And, how do we tell one form from another? With these fundamental questions in mind, the present paper presents a novel hypothesis:

State-specific forms of oxidative stress are associated with distinct protein cysteine redox patterns. The patterns:

I. Are encoded by the identity, amount, and redox vector determinants (chemotype, direction, magnitude) of specific proteins.
II. Evolve in space and time.
III. Display individuality at every level, from the organism to single cells.
IV. Are an expression of the state-specific form.
V. Can be functional.

In part 1, this treatise unfurls the vast complexity of the oxidative stress universe as a means to explore and elaborate the hypothesis. Part 2 weighs the evidence for cysteine redox patterns before part 3 discusses the implications for oxidative stress research. Of potentially profound importance, it may be possible to navigate the intricately nuanced and complex oxidative stress landscape by mapping constellation-like cysteine redox patterns as a guide to chart the roles ROS play in health and disease.

## 2 Part 1. The oxidative stress universe

### 2.1 50 shades of oxidative stress

**Fig. 1:**
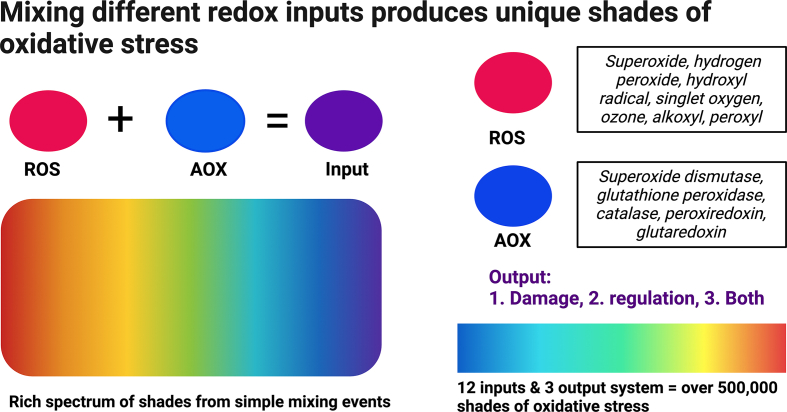
50 shades of oxidative stress. Like how mixing colours creates shades, the left side of the figure depicts how mixing discrete “ROS” and antioxidant (AOX) inputs can produce distinct “shades” of oxidative stress. The right side depicts how the elements of a simple 12 input and 3 output redox system can generate over half a million shades of oxidative stress. See the main text for specific details. (For interpretation of the references to colour in this figure legend, the reader is referred to the Web version of this article.)

In considering the potential number of specific oxidative stress forms, it is instructive to view ROS and antioxidants as primary colours that nature may mix, in different ways, to generate distinct “shades” of oxidative stress (see Fig. 1).

If we simply treat “ROS” (a) and “antioxidants” (b) as monolithic inputs, then we can derive 9 “shades” of oxidative stress as specified by the oxidative damage (c), redox-regulation (d), or both (e) outputs:

1. a = c
2. a = d
3. a = e
4. b = c
5. b = d
6. b = e
7. a + b = c
8. a + b = d
9. a + b = e

Including the eustress (*n* = 9), distress (*n* = 9), or neither (*n* = 9) outcome overlays, trebles the number to 27 shades of oxidative stress.

Introducing the biochemically defined entities sheltering beneath the “ROS” umbrella increases the number of shades. ROS include superoxide, hydrogen peroxide, singlet oxygen, hydroxyl radical, peroxyl radical, alkoxyl radical, and ozone [40]. If we exclude combinations (e.g., a: superoxide + hydrogen peroxide = c), then the 7 molecules yield 21 discrete forms of the ROS input (a). For example, #1 a: superoxide = c.

The above-mentioned ROS are arbitrarily included as a purely theoretical instrument to illustrate the oxidative stress space. Other inputs are possible. For example, another 7-species input module with greater overall specificity toward cysteine residues might be: hydrochloric acid, hydrogen peroxide, peroxynitrite, hydrogen sulfide, nitric oxide, peroxomonocarbonate, and lipid hydroperoxides. Hence, the ROS and antioxidant redox modules specified here are simply tools for highlighting the vast oxidative stress accessible to a minimal number of inputs.

What about antioxidants? If we set aside the complexities around what an “antioxidant” is [[40], [41], [42], [43], [44]] and confine our attention to just five enzymes (superoxide dismutase, glutathione peroxidase, catalase, peroxiredoxin, and thioredoxin), then we get 15 distinct shades of the antioxidant input (b). For example, #4 = b: glutathione peroxidase = c.

Even the minimal input (a: n = 7, b: n = 5, a + b = 12) and output (c, d, e = 3) integers yield 531,441 shades of oxidative stress. Adding the “reactive species interactome” inclusive of a veritable zoo-like menagerie of molecules [4,[45], [46], [47]], glutathione and every type of antioxidant including isoforms, and source (e.g., enzyme) and space (e.g., mitochondria) variables would expand the number of oxidative stress shades by orders of magnitude. The scale of the numbers derived from minimal inputs and outputs point to a vast potential biochemical oxidative stress space.

### 2.2 Painting different redox pictures in the oxidative stress box

**Fig. 2:**
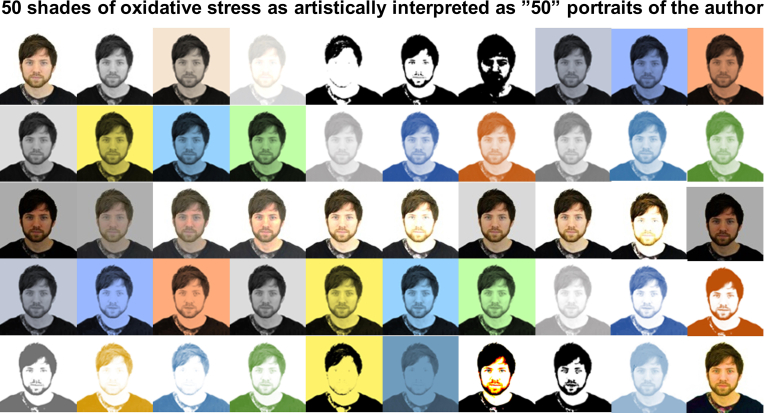
Painting different pictures in the oxidative stress box. The visual of the author's publicly available image, used with his permission, is represented in different ways that stress how subtly varying different inputs, in this case image saturation, contrast, and shading, can produce different outcomes. It is intended as a visual device to capture the essence of a hypothesis on distinct “shades” of oxidative stress. (For interpretation of the references to colour in this figure legend, the reader is referred to the Web version of this article.)

Before we delve deeper into the hypothesis, it may be helpful to visualise oxidative stress as a box. In essence, one could paint many different redox pictures, portraying specific forms of oxidative stress, within the canvas-like box (see Fig. 2). The kaleidoscope-like palette of shades, over half a million from minimal input-output integers, generate innumerable potential redox portraits. In the next section, I consider the cysteine redox canvas the forms may be painted upon.

### 2.3 The cysteine redox canvas

**Fig. 3:**
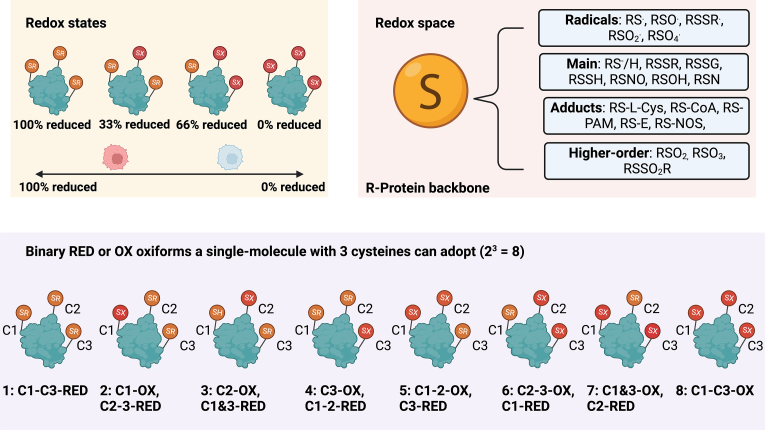
A visual overview of the cysteine landscape. Redox states. A single-molecule with 3-cysteines can adopt one of 4 mathematically permitted by the residue integer percentage cysteine redox states. At the population level, such as the two single cells depicted, the total pool of the protein identity specified single molecule can fall anywhere on a 0–100% redox state spectrum. Redox space. The sulfur atom in cysteine can adopt a panoply of different chemotypes. They are grouped by their character, such as free radical chemotypes like thiyl radicals. The redox space presented is not exhaustive. Oxiforms. According to a quadratic *nR* law whereby *n* is the redox state (2, reduced or oxidised) and *r* is the residue integers, respectively, a single-molecule with 3-cysteines can adopt 8 unique oxiforms. They distribute unevenly by the cysteine redox state: 100 (1), 33 (3), 66 (3), 0 (1).

After considering how redox “colours” produce distinct “shades” of oxidative stress, let us describe the cysteine redox canvas. The features of the cysteine redox landscape (see Fig. 3) are vast:

1. **Elements.** The elements of a cysteine proteome include the number of residues, proteins, and their copy numbers. To get an idea of the numbers involved, the human genome encodes over 200,000 cysteine residues distributed across somewhere in the order of 18,000 proteins [[48], [49], [50]]. The number of protein-specific single-molecules in a cell, can vary by 9, from 0 to millions, orders of magnitude [[51], [52], [53]].
2. **Redox space.** From 8 formal oxidation states (e.g., persulfide = sulfur +1), the sulfur atom can assume multiple distinct chemotypes inclusive of over 20 post-translational modifications (PTMs) [31,54,55]. A chemotype can alter the functional properties of the modified protein [56]. For instance, cysteine sulfenic acids (sulfur = 0) are soft electrophiles and weak nucleophiles.
3. **Redox state.** The permitted percentage cysteine redox state of a single-molecule is given by the binary reduced or oxidised state of each residue. To qualify permissible, a single-molecule with 1 cysteine can only be 0 or 100% oxidised. Residue integers govern the mathematically allowed percentage graded classes (e.g., 4-cysteines = 5 classes [0, 25, 50, 75, 100]). The number of single-molecules in each percentage grade yields the classed-averaged redox state of the population. If there were 10,000 copies of a protein with 1 cysteine in a cell, then the class average value can fall anywhere on a percentage spectrum. For example, 1,000–100% oxidised single-molecules = 10% oxidised. The global redox state of the cysteine proteome, typically 15-25%-oxidised [57], is set by the weighted mean of all the protein-specific sums.
4. **Proteoforms.** With the residues and chemotypes as building blocks, it is possible to generate manifold unique cysteine proteoforms [[58], [59], [60]] called oxiforms [61]. If there are 20 sulfur chemotypes, then the 100%-oxidised state for a single-molecule with 1 cysteine includes 20 unique oxiforms. Oxiforms scale with the residue integer according to a quadratic law. For example, in binary reduced or oxidised terms, a single-molecule with 10 cysteines can adopt 1,024 unique residue-defined oxiforms distributed across 11 percentage grades (e.g., 10% oxidised = 10 possible oxiforms).

To get a sense of the epic scale of the canvas, consider 10 discrete proteins with 1 cysteine (less than 0.005% of the cysteine proteome). Their whole-integer percentage redox state alone gives 1 x 10^20^ possible combinations (e.g., 1 all 10 are 0%-oxidised). For context, the estimated number of stars in the universe is 7 x 10^23^. Simplifying matters by restricting the number of combinations to a narrow 5% zonal space, gives 100000 possible combinations. The evidence-based lines of scientific reasoning presented suggest ample space for, if only purely theoretically, patterning shaded oxidative stress pictures onto the cysteine redox canvas.

### 2.4 The cysteine redox pattern hypothesis

**Fig. 4:**
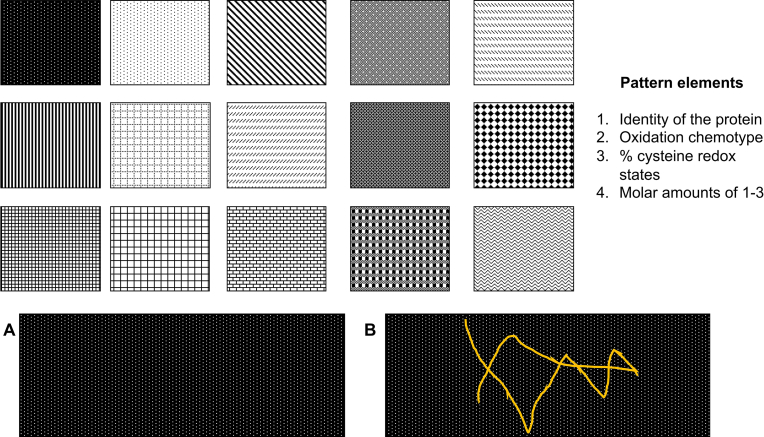
The cysteine redox pattern hypothesis: a visual representation. In essence, the basic pattern elements, listed 1–4, can be combined in myriad of ways to translate unique redox inputs into specific patterns of oxidised proteins. Although the geometries portrayed here are purely arbitrary, the hypothesis predicts the existence of patterns that can be geometrically represented by the distance between individual elements and in, 3 and 4D, the magnitude of the connecting line, as encoded by percent cysteine oxidation, over time. At the bottom, panel A shows as individual dots a vast array of possibilities encoded by a defined subset of the cysteine proteome. Panel B uses a squiggled line to show the potential for a specific pattern associated with a unique form or shade of oxidative stress to be drawn onto the vast canvas. (For interpretation of the references to colour in this figure legend, the reader is referred to the Web version of this article.)

The information presented contextualises the present papers hypothesis that: state-specific forms of oxidative stress are associated with distinct protein cysteine redox patterns (see Fig. 4). For example, the hypothesis predicts that a given state, such as exercise, is associated with a specific cysteine redox pattern, which sets it apart from other states, such as rest [[62], [63], [64]]. The patterns:

I. Are encoded by the identity, amount, and redox vector determinants (chemotype, direction, magnitude) of specific proteins.
II. Evolve in space and time.
III. Display individuality at every level, from the organism to single cells.
IV. Are an expression of the state-specific oxidative stress form.
V. Can be functional.

**Fig. 5:**
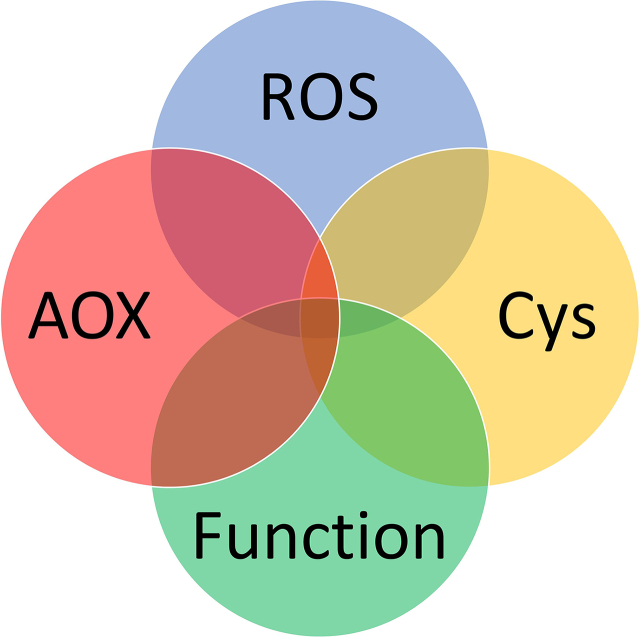
A schematic illustrating the underlying conceptual model. In this model, a redox input comprising the intersection of the ROS and antioxidant (AOX) generates a specific cysteine output, in the form of a pattern. The pattern may link in a casually important way to a functional outcome, such as a disease state. This relationship being visually represented at the nexus of the Venn diagram.

The hypothesis is grounded in an integrative conceptual model. To explore it, let us step outside the oxidative stress box and imagine 4 circles (see Fig. 5). Circle 1 and 2 cover specific “ROS” and “antioxidant” inputs. Like how colours mix to produce shades, they coalesce to produce a redox input signature [65]. Or “shade” of oxidative stress. Circle 3 frames the pattern the shade of oxidative stress paints onto the cysteine redox canvas of the cell. All the circles can input into a “functional” output-generating module: circle 4. As discussed in detail below, the output can be functionally connected to the state or simply be a product of it.

The importance of cysteine oxidation means, in some cases, the functional output is the pattern itself or a specific part thereof. That is, circle 3–4 can be one and the same. When a given cysteine oxidation event isn't functional in the classic sense of the word, such as inactivating an enzyme, it plays some wider role, however minor, by consuming a species and, when it is reduced, NADPH. That is, the totality of the cysteine proteome might be an ‘antioxidant’ [66]. For example, consuming a species may prevent a reaction with another target. Perhaps, preventing hydrogen peroxide from reacting with a redox-active metal ion to produce hydroxyl radical, or a species that resembles it [67]. The functional impact of many such events on a proteome-wide scale may be appreciable.

## 3 Part 2. The evidence

Having expressed the conceptually-grounded hypothesis, let us weigh the evidence. Evidence relating to the redox inputs producing the patterns and other relevant aspects is presented separately (see Table 1). The present hypothesis makes testable predictions. One is that: a distinct redox input generates a specific cysteine redox output, such as oxidising discrete proteins. Two discrete redox inputs producing the same cysteine redox output would violate the hypothesis.

**Table 1:** Selected theoretically principled points related to the redox input.

Point	Example	Implication for patterns
*Immutable differences in the species-specific chemistry*	Unlike superoxide, hydroxyl radical can directly oxidise guanine bases in DNA at an appreciable rate.	The patterns will be shaped by the profile, such as type, of ROS produced.
*Graded commonalties in the species-specific chemistry between individual ROS*.	Many ROS, from superoxide to hydroxyl radical, react with cysteine but the rate constants and reaction products differ. Hence, common targets with graded reactivities.	Cysteine is a common “funnel” like point for distinct ROS inputs. Variable input-specific reaction mechanisms and preferences will be reflected in the cysteine chemotypes formed and the identity of the oxidised targets.
*Graded commonalties in the species-specific chemistry within individual ROS*.	The kinetic rate constant for the 2nd order biomolecular reaction between hydrogen peroxide and the catalytic cysteines in peroxiredoxin 2 and PTP1B differ by orders of magnitude.	Kinetics influences the patterns by shaping the identity of the proteins oxidised and the magnitude of their oxidation.
*Antioxidants are unique.*	Superoxide dismutase reacts with the superoxide anion at an appreciable rate but not the protonated hydroperoxyl radical.	The antioxidant make-up of a cell or compartment shapes the pattern.
*Antioxidant enzyme activity is influenced by copy number and post-translational modifications*	The activity of superoxide dismutase 2, the mitochondrial isoform, can be decreased on electrostatic grounds by lysine acetylation.	The pattern will be determined by the influence of the active antioxidants in a redox niche on the ROS input.
*Certain antioxidant enzymes directly control cysteine oxidation.*	Peroxiredoxin 2 transfers hydrogen peroxide derived electrons to STAT3 in a relay type mechanism [68].	Active antioxidants in a redox niche can convert ROS inputs into cysteine oxidation outputs.
*The bilateral metabolism principle*	Ultimately, the redox input is controlled by the NAD^+^/NADH and NADP^+^/NADPH available to “fuel” the relevant reactions.	Metabolism shapes the cysteine oxidation pattern at almost every level.

### 3.1 There are cysteine redox patterns

Using bottom-up mass spectrometry (m/s) [[69], [70], [71]], Jones group [72] found that unique cysteine containing peptides were oxidised when the thioredoxin (input 1) compared to the glutathione (input 2) systems were chemically inhibited in cells. When the two inputs oxidised the same peptide, the cysteine redox vector, the magnitude of the observed oxidation, usually differed. Their work and other studies (see Table 2) comparing discrete states [[73], [74], [75], [76], [77], [78], [79], [80], [81]], from rest vs. exercise in humans [82] to night vs. day in cyanobacteria [83], support input-specific cysteine redox state outputs.

**Table 2:** Selected m/s-based examples of distinct cysteine redox differences between divergent inputs.

REF	States	Summary
83	*Light vs. day in cyanobacteria*	**Magnitude pattern**: Hundreds of the same cysteine residues went from 5 to 20% oxidised in the light to 21–40% in the dark.
82	*Young vs. old human skeletal muscle at rest*	**Reductive pattern**: Multiple cytosolic cysteines were more reduced in old vs. young individuals.
75	*Acute vs. chronic oxidative stress in mice*	**Identity pattern**: Limited similarities in the nature of the proteins and residues oxidised in chronic vs. acute oxidative stress models.
79	*Chemotype profiling of different modifications in mouse liver*	**Chemotype patterns**: Analysis of 10^3^ sites revealed little overlap between different modifications.
76	Comparison of hydrogen peroxide vs. hypochlorite induced oxidative stress in bacteria.	**Identity pattern**: Some proteins are preferentially oxidised by hydrogen peroxide vs. hypochlorite.

As Murphy reviews [8], mitochondrial superoxide production is governed by a universal equation shaped by enzyme and site-specific variables. Consider complex I. While the exact site is disputed [84,85], the properties of forward electron transfer (FET) vs. reverse electron transfer (RET)-dependent superoxide production differ [[86], [87], [88], [89], [90], [91]]. In general, FET defines a more durable shallow slow-release mode compared to the brief high-amplitude burst-like character of RET. Dröse group [92] showed how FET and RET-induced superoxide production in isolated mitochondria oxidised distinct proteins. The fold-change values of commonly oxidised proteins differed. They revealed, in line with the different release topology [93], that complex III-induced superoxide production oxidised distinct targets. Their findings suggest generator-specific cysteine oxidation patterns. Hence, circles 1–3 of the conceptual model grounding the present hypothesis seem experimentally connected.

The Chouchani [94] group synthesised non-hydrolysable cysteine reactive phosphate tags (CPTs) to selectively enrich oxidised peptides. They achieved the deepest cysteine m/s coverage ever recorded. CPTs identified 171,000 unique oxidised peptides and stoichiometrically quantified the cysteine redox state of 34,000 distinct sites distributed across 9,000 proteins in multiple tissues harvested from young and old mice. Among the many illuminating findings, two stand out. First, the cysteine redox landscape displayed tissue-specific patterns. Second, ageing influenced the cysteine oxidation patterns in a tissue-specific manner. Overall, the available evidence supports the idea of cysteine oxidation patterns.

### 3.2 Cysteine oxidation patterns evolve in space and time

The latest insights [95,96] support the spatial patterning of the cysteine redox landscape by cellular nanodomain [97] into heterogenous, potentially phase-separated, niches [2,[98], [99], [100], [101]]. The spatially resolved hubs grow organically from the discrete production and consumption of specific ROS by the redox apparatus inhabiting each niche, as the generator-specific ROS example attests [92]. Evidence suggests cysteine oxidation evolves on the timescales of seconds to hours (e.g., signalling [102,103]), days (e.g., circadian rhythms [[104], [105], [106]]), months (e.g., the menstrual cycle [107]), and across years (e.g., ageing). Overall, principled lines of scientific reasoning drawn from the cysteine oxidation literature support spatiotemporal cysteine oxidation patterning.

### 3.3 Cysteine oxidation patterns display individuality at every level

Based on the pioneering insights of Nikolaidis [108,109], Margaritelis [110] and others [110], the third age of redox research is set to witness the rise of personalised redox biology. Given understanding of redox heterogeneity is emerging, the discussion will necessarily be brief. Suffice to say, the hypothesis predicts individuality in all its forms and levels, from single-molecule proteoforms to organisms. For example, burgeoning evidence around the biological variability of ROS levels between cells and the proteome of each cell implies heterogenous cysteine oxidation in single cells [[111], [112], [113], [114], [115], [116], [117]]. It is the aggregate cysteine oxidation pattern of millions of cells that gives rise to tissue-specific patterns. In line with recent evidence [118], one would predict biological sex to shape state-specific cysteine oxidation patterns.

### 3.4 Functional aspects

A state-specific cysteine oxidation pattern would be an expression of the same, but would the pattern be functional? Certainly, the functionality of cysteine oxidation is a foundational tenet of redox-regulation [[119], [120], [121], [122], [123]]. At the single-molecule level, cysteine oxidation can completely control a given parameter. Take GAPDH (UniProt:P04406). Nucleophilic Cys152 catalysis requires the reduced deprotonated cysteine. Cys152 oxidation to, for example, a sulfenic acid inactivates the enzyme on electrophilic grounds [[124], [125], [126]]. On a scale of 0–1, with the former denoting no and the latter total control, we can state that Cys152 redox state fully (i.e., 1) controls the enzyme activity of the single-molecule.

Extending our single-molecule example to the population level complicates matters. The number of GAPDH molecules in the population, such as an SC, may buffer the impact of cysteine oxidation on enzyme activity and thereby glycolytic flux. The influence of cysteine oxidation on glycolytic flux becomes a mobile element capable of falling anywhere between 0 and 1. For instance, the functional impact of oxidising 10% of the total GAPDH molecules in the population may be buffered (e.g., weight = 0.1) by the remaining 90% of reduced GAPDH molecules until a critical threshold is surpassed. Threshold integers would vary based on the total number of GAPDH molecules and outcome co-dependents in the cell. The same thinking applies to the tissue level via the aggregates of millions of individual SC readouts. Many checks and balances, such as phosphorylation, influence functional effects. Still, GAPDH oxidation can influence a phenotype, such as safeguarding the reductive capacity of stressed tumour cells to support their survival [127].

**Fig. 6:**
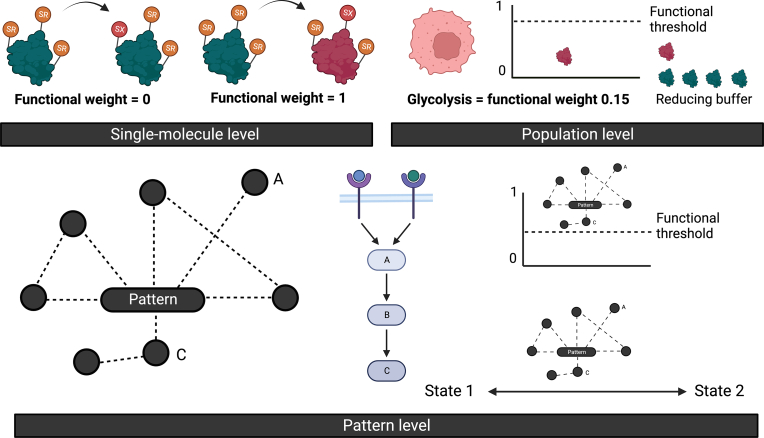
Visual overview of selected functional aspects. Single-molecule level. Left and right scheme show a residue-specific cysteine event, reduced to oxidised transition, having 0 and 1 functional weight on a defined parameter, such as enzyme activity, respectively. Population level. The panel shows the functional impact, even for a 1-weighted single-molecule effect, of a small number of oxidised molecules being buffered by the majority of reduced molecules. Pattern level. The panel shows a pattern and relates the nodes A and B to receptor-linked convergent biological pathway. In this case, the weighted effect of the pattern surpasses a functional threshold. As a result, there is a causal transition from state 1 to state 2.

The state of affairs (see Fig. 6) become infinitely more complex, when we shift our thinking from the impact of one protein on a process to the aggregated functional weight of a multiparametric pattern on a phenotype. For most states, the functional weight of the specific cysteine oxidation pattern is extremely unlikely to be binary (i.e., 0 or 1). From first principles, one might predict the oxidation of many redox-regulated proteins responsible for shaping the outputs of different pathways in a hub-like manner to display a degree of granular functionality over the state. We might take some encouragement from how blanketing cells with the “antioxidant” N-acetylcysteine [128] has, and presumably by acting on multiple proteins, negated certain ROS-regulated growth-factor phenotypes [25].

Assigning an exact functional weight to a specific cysteine oxidation pattern is difficult. It goes beyond back of the envelope maths summing the different functional weights of individual nodes, especially when the function of many oxidation events is unknown. Functional weights would also exhibit marked individuality. The rise of advanced artificial intelligence inspired computational capabilities and mathematical redox modelling suggests a promising path forward [[129], [130], [131], [132], [133], [134], [135], [136], [137]]. In the end, one must manipulate a pattern to appraise its functional weight. For example, using an antioxidant to manipulate exercise-induced cysteine oxidation by measuring molecular adaptations as a functional readout [[138], [139], [140], [141], [142], [143]].

Overall, it is difficult to ascribe a precise functional value to the holistic output of a state-specific cysteine redox pattern with respect to being causally related in a meaningful way to said state.

## 4 Part 3. Discussion

Having presented the hypothesis and weighed the evidence, let us succinctly discuss what it means for oxidative stress research.

### 4.1 Measuring

Most of the fraught and often binary “yes or no” (monochrome) answers for measuring oxidative stress [[144], [145], [146], [147], [148], [149], [150]] are ill-suited to telling one shade apart from another. For instance, DNA oxidation tends to go up in many different diseases. And so, the technological means to measure state-specific oxidative stress encoded by a tell-tale cysteine oxidation pattern would be an invaluable research tool. A tool to biochemically define specific states would progress toward a systems redox understanding of emergent complexity. It could advance understanding of oxidative stress in all its forms by discriminating specific biochemical states, helping to reveal their biological basis with respect to their redox signature origins, and provide a mechanism to unmask individuality. Like the chances of locating an electron in a probability cloud, I would expect to find most individuals, be it an SC or person, within the bounds of a specific region. The outliers may hold the key to unlocking new, upending, and potentially functionally significant mechanisms of state-specific oxidative stress. A quantitative tell-tale state-specific readout would allow to unmask its origins, such as the specific ROS source, by complementing the rich analytical toolbox for measuring ROS [[151], [152], [153], [154], [155]], antioxidants [[156], [157], [158], [159]], and phenotypes [[160], [161], [162], [163], [164]].

### 4.2 Manipulating

Chief among the complexities of defining specific roles for ROS, especially in disease, is the mechanistic requirement for the manipulation, usually an antioxidant, to modify oxidative stress [165]. And to selectively modify the negative aspects without meddling with beneficial effects; which would typically mean not sacrificing redox-regulation on the altar of reducing oxidative damage. This would also likely involve reversing an aberrant cysteine oxidation pattern, perhaps the increased oxidation of specific proteins [166], to “healthy” state. For instance, restoring an aged pattern in skeletal muscle toward a younger one [93,167,168]. A state-specific cysteine oxidation pattern would be instrumental for appraising whether a given manipulation, such as manganese porphyrins [[169], [170], [171], [172], [173]], satisfies essential mechanistic criteria. So doing, would help close chasm-like gaps in our current understanding of if and how antioxidants actually modified oxidative stress as it manifested in a state-specific context.

### 4.3 Interpreting

Together with the functional ones expressed already, some interpretational points are worth considering:

1. **Trajectories.** Like ripples in the water radiating from an initial splash, a state triggering pattern may evolve to a different, even unrecognisable, form due to the probable substantial lag between the origins of a disease and its onset. In such cases reverse engineering let alone observing the starting point from the observed trajectory point would be near impossible. Still, measuring evolving patterns may provide important insights into disease progression.
2. **Boundaries.** In some cases, it may be difficult to pinpoint one moment in time demarcating a state change, such as a “healthy” brain to an Alzheimer's one [[174], [175], [176], [177]]. The boundary lines demarkating the states would blur. Blurred lines draw attention to the technological need to cover much of the vast pattern-generating space.
3. **Specificity.** A state may be an amalgam of patterns covering specific tissues. With no one form representing an absolute and definitive organism level pattern. Hence, a distinct tool would be needed to measure specific patterns in a tissue or systemic sample. Such a need may pose challenges should there be no or a blurred systemic pattern of a tissue-originated phenotype.

### 4.4 Technologies

As the OxiMouse study attests [93], technological breakthroughs inspire boundary pushing advances. More technological “quantum-leaps” are needed to fully explore the present hypothesis and its implications for oxidative stress. Major bottlenecks define the need for sophisticated technologies to digitally quantify oxiforms with residue and chemotype information in a targeted way and in an unbiased proteome-wide manner. For example, a pattern might be encoded by the number of redox-class defined oxiforms. Unmasking it demands single-molecule analysis. However, current oxiform technologies, such as immunoblotting with ectopic mobility-shifting payloads, are generally ineffective at detecting oxiforms per se let alone digitally quantifying them [[178], [179], [180], [181], [182], [183]].

Exciting advances on several fronts, from chemotype probes [[184], [185], [186], [187]], m/s technologies [[188], [189], [190], [191], [192], [193]], and next-generation sequencing [[194], [195], [196], [197], [198]] may surmount many current technical bottlenecks. Next-generation sequencing technology inspired advances [[199], [200], [201], [202]] in fluorescent redox immunoassays, such as RedoxiFluor [[203], [204], [205], [206]], would meet the need to digitally quantify multiple pattern-generating residue-resolved oxiforms in a massively parallel way on a portable machine. The resultant implications for point of care oxidative stress testing would be profound. In time, concerted technological advances will better allow to identify and quantify state-specific oxidative stress encoded by cysteine oxidation patterns.

## 5 Conclusion

The present paper posits that the biochemically complex phenomenon of oxidative stress can be understood through the lens of distinct cysteine oxidation patterns. These patterns, influenced by a multitude of factors, encode unique oxidative stress signatures associated with specific states, such as a disease. While the evidence supporting such patterns is promising, further research, and pioneering technologies are needed to fully unravel their complexities and functional significance.

Measuring and manipulating these patterns promises to advance our understanding of oxidative stress in health and disease. They offer a nuanced framework for dissecting the intricate biochemistry underlying state-specific oxidative stress. Exploring the hypothesis may uncover novel insights about state-specific oxidative stress, ultimately paving the way for more targeted and effective redox-active interventions.

## Use of AI

Artificial intelligence, such as ChatGPT, did not write the manuscript.

## Declaration of competing interest

There are no conflicts of interest to declare.

## Data Availability

No data was used for the research described in the article.
